# Evidence conflict measure based on OWA operator in open world

**DOI:** 10.1371/journal.pone.0177828

**Published:** 2017-05-18

**Authors:** Wen Jiang, Shiyu Wang, Xiang Liu, Hanqing Zheng, Boya Wei

**Affiliations:** 1 School of Electronics and Information, Northwestern Polytechnical University, Xi’an, Shaanxi Province, 710072, China; 2 Infrared Detection Technology Research & Development Center, Shanghai Institute of Spaceflight Control Technology, CASC, Shanghai 200233, China; 3 Shanghai Institute of Spaceflight Control Technology, Shanghai 200233, China; Southwest University, CHINA

## Abstract

Dempster-Shafer evidence theory has been extensively used in many information fusion systems since it was proposed by Dempster and extended by Shafer. Many scholars have been conducted on conflict management of Dempster-Shafer evidence theory in past decades. However, how to determine a potent parameter to measure evidence conflict, when the given environment is in an open world, namely the frame of discernment is incomplete, is still an open issue. In this paper, a new method which combines generalized conflict coefficient, generalized evidence distance, and generalized interval correlation coefficient based on ordered weighted averaging (OWA) operator, to measure the conflict of evidence is presented. Through ordered weighted average of these three parameters, the combinatorial coefficient can still measure the conflict effectively when one or two parameters are not valid. Several numerical examples demonstrate the effectiveness of the proposed method.

## Introduction

With the rapid development of industry and military, the speed and quantity of information acquisition are growing at a rapid rate, and various demands emerge increasingly based on the collected information, such as opinion exchange [1] and object assessment [2]. At the same time, the obtained information is often uncertain because of its high complexity, susceptibility to interference, and the malicious effects of the enemy in military and radar information [3, 4]. Dempster-Shafer evidence theory (D-S theory), proposed by Dempster [5] and extended by Shafer [6], is one of the common methods used to solve the problem of uncertainty. As compared with probability theory, D-S theory uses the confidence interval instead of the specific probability value to describe the possibility and could be able to handle uncertainty issues more effectively; as the generalized Bayesian theory [7–9], D-S theory can deal with the evidence when a priori information is not clear. Based on these advantages, D-S theory has been widely applicated in many systems such as risk and reliability analysis [10–13], decision making [14–18], classifier recognition [19–22], diagnosis fault [23, 24], and uncertain information processing [25–32].

After Dempster’s combination rule is proposed by Shafer [6], information from different sources can be simply combined. Thereby D-S theory is widely used in information fusion [33–39]. But, Zadeh [40] put forward a paradox about information fusion, which showed that directly using Dempster’s combination rule resulted in a fallacious result in the case of highly conflict evidence. Subsequently, many scholars improved the combination rule, such as Yager’s [41] method and Smets’s [42] method. Lefèvre [43] finally summed up a unified combination formula of variable coefficients, it may contain several formulas previously proposed. However, shortly after this paper was published, Haenni [44] refuted his argument and proposed a novel idea of dealing with conflicting evidence by modifying the model. Murphy [45] put forward the idea of combining the average evidence. Jousselme [46] proposed the concept of evidence distance. A series of methods about modifying the evidence and conflict management have been published [47–51].

To solve conflict management problem, Deng [52] proposed generalized evidence theory (GET). GET concludes that there are two main causes for evidence conflicts. One is questionable sensor reliability caused by disturbances or the condition of equipment. The other is that the system is in open world where our knowledge is not complete. In comparison with classical D-S theory, GET addresses conflict management in an open world, where the frame of discernment is incomplete because of uncertainty and incomplete knowledge. Thereby GET is able to handle the conflict between the evidence in open world. GET cancel the restrictions on the *m*(∅) = 0 in D-S theory. That is, there may exist some hypotheses or propositions beyond the fixed frame of discernment. The value of *m*(∅) indicates the open world degree of the frame of discernment. GET not only inherits the advantages of classical D-S theory, but also further expands the superiority of D-S theory in solving uncertainty issues [53].

Although GET is a powerful and effective promotion of D-S theory, there is still no efficacious method to measure the conflict between generalized evidence. How to define a reasonable variable to describe the conflict, is a problem worthy of studying. Liu [54] proposed using a two-dimensional parameter to measure the conflict, which broke the shackle of using a single coefficient. Moreover, a method by using the two-tuple of *k* and evidence distance to describe the conflict in open world is presented in [52]. These idea are both measure the conflict by two different parameters, but their respective applicable occasions must be analyzed first which greatly reduces the efficiency. In real application, a measure value of conflict is often needed. Based on the mentioned above, a new coefficient *gir* called generalized interval correlation coefficient, which is extended from the interval correlation coefficient [55], is proposed in this paper. Then we propose a method which combines generalized conflict coefficient, generalized evidence distance, and generalized interval correlation coefficient based on the ordered weighted averaging (OWA) operator [56], to measure the conflict of evidence in open world.

The remainder of this paper is organized as follows. Section “Preliminaries” starts with a brief presentation of D-S theory and some other necessary related concepts. In Section “Generalized interval correlation coefficient”, we present the generalized interval correlation coefficient. Then we propose a new method to measure the conflict based on OWA operator and discuss its application in Section “A combinatorial parameter based on OWA operator”. Conclusions are summarized in Section “Conclusion”.

## Preliminaries

### Dempster-shafer evidence theory

Dempster-Shafer evidence theory, as introduced by Demster [5] and expanded later by Shafer [6], is used to deal with the proposition of uncertainty.

Θ is defined as a sample space, which is called a frame of discernment (FOD). Θ by the state of things might composition, and these states are mutually exclusive and exhaustive.

$$\begin{array}{c} \Theta =\{\emptyset ,H_{1},H_{2},\cdots ,H_{i},\cdots ,H_{N}\}. \end{array}$$ (1)

Let 2^Θ^ be the set of all subsets of Θ, namely

$$\begin{array}{c} 2^{\Theta }=\left\{\emptyset ,H_{1},H_{2},\cdots ,H_{N},\left\{H_{1}\cup H_{2}\right\},\cdots ,\left\{H_{1}\cup H_{2}\cup \cdots \cup H_{i}\right\},\cdots ,\Theta \right\}. \end{array}$$ (2)

2^Θ^ is called the power set of Θ. For FOD Θ, a mass function is a mapping *m* from 2^Θ^ to [0, 1], formally defines as

$$\begin{array}{c} m:2^{\Theta }\to \left[0,1\right], \end{array}$$ (3)

it is also called the basic probability assignment (BPA). BPA must satisfies the following condition

$$\begin{array}{ccc} \sum_{A\in 2^{\Theta }}m\left(A\right) & = & 1,\\ m(\emptyset ) & = & 0. \end{array}$$ (4)

For a BPA, the belief function *Bel*: 2^Θ^ → [0, 1] is defined as

$$\begin{array}{c} Bel\left(A\right)=\sum_{B\subseteq A}m\left(B\right), \end{array}$$ (5)

the plausibility function *Pl*: 2^Θ^ → [0, 1] is defined as

$$\begin{array}{c} Pl\left(A\right)=1-Bel\left(\bar{A}\right)=\sum_{B\cap A\neq \emptyset }m\left(B\right). \end{array}$$ (6)

For two BPAs *m*_1_ and *m*_2_ that have been given, they can be combined by Dempster’s combination rule.

$$\begin{array}{c} m\left(A\right)=\frac{1}{1-k}\sum_{B\cap C=A}m_{1}\left(B\right)m_{2}\left(C\right), \end{array}$$ (7)

where

$$\begin{array}{c} k=\sum_{B\cap C=\emptyset }m_{1}\left(B\right)m_{2}\left(C\right). \end{array}$$ (8)

k is between [0, 1], named as the coefficient of conflict, is a conflict measurement between two pieces of evidence. The larger the value of *k*, the greater the conflict between the two evidence. When *k* = 1, Dempster’s combination rule will be invalid. The combination rule provides precious properties, as the commutativity and the associativity, thus it is able to dispose multiple pieces of evidence expediently.

### Generalized evidence theory (GET)

Deng [52] proposed GET, which is extended by D-S theory, to solve the problems in incomplete frame of discernment. GET eliminates the *m*(∅) = 0 constraint in the Dempster-Shafer evidence theory. The physical meaning of *m*(∅) is used to measure the qualities that are not contained in the FOD. It is prerequisite to limit it to zero because the FOD defined in D-S theory is exhaustive and exhaustive. But with the deepening use of the D-S theory, resulting in a growing number of open world problems, that is obtained FOD is incomplete at this time. Assigning unrecognized mass to *m*(∅) helps handle the open world problems.

Suppose that *U* is a frame of discernment in an open world. Its power set, $2_{G}^{U}$, is composed of 2^*U*^ propositions, ∀*A* ⊂ *U*. A mass function is a mapping $m_{G}:2_{G}^{U}\to \left[0,1\right]$ that satisfies

$$\begin{array}{c} \sum_{A\in 2_{G}^{U}}m_{G}\left(A\right)=1. \end{array}$$ (9)

So, *m*_*G*_ is the generalized basic probability assignment (GBPA) of the FOD *U*. When *m*_*G*_(∅) = 0, the GBPA degenerate into a traditional BPA. Given a GBPA *m*, GBel is defined as

$$\begin{array}{ccc} GBel(A) & = & \sum_{B\subseteq A}m\left(B\right),\\ GBel(\emptyset ) & = & m(\emptyset ). \end{array}$$ (10)

GPl is defined as

$$\begin{array}{ccc} GPl(A) & = & \sum_{B\cap A\neq \emptyset }m\left(B\right),\\ GPl(\emptyset ) & = & m(\emptyset ). \end{array}$$ (11)

Given two GBPAs *m*_1_ and *m*_2_, the generalized combination rule (GCR) satisfies

$$\begin{array}{c} m\left(A\right)=\frac{\left(1-m\left(\emptyset \right)\right)\sum_{B\cap C=A}m_{1}\left(B\right)m_{2}\left(C\right)}{1-K}, \end{array}$$ (12)

with

$$\begin{array}{ccc} K & = & \sum_{B\cap C=\emptyset }m_{1}\left(B\right)m_{2}\left(C\right),\\ m(\emptyset ) & = & m_{1}\left(\emptyset \right)m_{2}\left(\emptyset \right),\\ m(\emptyset ) & = & 1,\;\;\text{if}\;\text{and}\;\text{only}\;\text{if}\;K=1. \end{array}$$ (13)

### Jousselme’s evidence distance

Jousselme [46] proposed a new distance to measure the difference between two bodies of evidence, which is also called the evidence distance.

Let *m*_1_ and *m*_2_ be two BPAs on the same frame of discernment Θ, which contain *N* mutually exclusive and exhaustive hypotheses. Let *d*_*BPA*_(*m*_1_, *m*_2_) represent the distance between *m*_1_ and *m*_2_, defined as

$$\begin{array}{c} d_{BPA}\left(m_{1},m_{2}\right)=\sqrt{\frac{1}{2}{\left(\vec{m_{1}}-\vec{m_{2}}\right)}^{T}\underline{\underline{D}}\left(\vec{m_{1}}-\vec{m_{2}}\right)}, \end{array}$$ (14)

where *m*_1_ and *m*_2_ are two BPAs. And $\underline{\underline{D}}$ is a 2^*N*^ × 2^*N*^ matrix whose elements are $\underline{\underline{D}}\left(A,B\right)=\frac{|A\cap B|}{|A\cup B|}$, where *A*, *B* ∈ *P*(Θ) are derived from *m*_1_ and *m*_2_, respectively.

And Deng [52] extended it to the generalized evidence distance. The generalized evidence distance *dis* between *m*_1_ and *m*_2_ is defined as

$$\begin{array}{c} d_{GBPA}\left(m_{1},m_{2}\right)=\sqrt{\frac{1}{2}{\left(\vec{m_{1}}-\vec{m_{2}}\right)}^{T}\bar{D}\left(\vec{m_{1}}-\vec{m_{2}}\right)}, \end{array}$$ (15)

where *m*_1_ and *m*_2_ are two GBPAs. And $\bar{D}$ is a 2^*N*^ × 2^*N*^ matrix whose elements are $\bar{D}\left(A,B\right)=\left\{ \begin{array}{cc} \frac{|A\cap B|}{|A\cup B|} & \text{if}\;A,B\;\text{are}\;\text{not}\;\text{all}\;\emptyset ,\\ 1 & \text{if}\;A=B=\emptyset . \end{array}\right.$

### Interval correlation coefficient

Wu [55] proposed a new coefficient to describe the correlation between the two evidence.

Let *m*_1_ and *m*_2_ be two BPAs on the same frame of discernment Θ = {*θ*_1_, *θ*_2_, …, *θ*_*N*_}. It is expressed as a confidence interval of all the single subset of elements on the frame of discernment. That is, *m*_1_ and *m*_2_ is expressed as [*Bel*_1_(*θ*_*i*_), *Pl*_1_(*θ*_*i*_)], *i* = 1, 2, …, *N* and [*Bel*_2_(*θ*_*i*_), *Pl*_2_(*θ*_*i*_)], *i* = 1, 2, …, *N*. Let *ir*(*m*_1_, *m*_2_) represent the interval correlation coefficient between two BPAs *m*_1_ and *m*_2_, defined as

$$\begin{array}{c} ir\left(m_{1},m_{2}\right)=\frac{c(m_{1},m_{2})}{\sqrt{c\left(m_{1},m_{1}\right)\times c\left(m_{2},m_{2}\right)}}, \end{array}$$ (16)

where

$$\begin{array}{c} c\left(m_{a},m_{b}\right)=\frac{1}{2}\sum_{i=1}^{N}\left(Bel_{a}\left(\theta_{i}\right)Bel_{b}\left(\theta_{i}\right)+Pl_{a}\left(\theta_{i}\right)Pl_{b}\left(\theta_{i}\right)\right),\;\;a,b=1,2 \end{array}$$ (17)

### Ordered weighted averaging (OWA) operator

Averaging operator is a tool to implement information fusion [57, 58]. Yager [56] proposed an OWA operator that is tantamount to the “or” operation and “and” operation to be extended in fuzzy operation. When the operator is applied, its “and or” degree can be adjusted according to specific requirement, thereby more able to meet the practical application needs.

Suppose *F*: *R*^*n*^ → *R*, there is an n-dimensional weight vector *ω* associated with *F*, *ω* = (*ω*_1_, *ω*_2_, …, *ω*_*n*_), *b*_*i*_ is the i-th largest element in array (*a*_1_, *a*_2_, …, *a*_*n*_). N-dimensional OWA operator *F* is defined as

$$\begin{array}{c} F\left(a_{1},a_{2},\cdots ,a_{n}\right)=\sum_{i=1}^{n}\omega_{i}b_{i}, \end{array}$$ (18)

which satisfies the following condition

$$\begin{array}{ccc} & & \omega_{i}\in \left[0,1\right],\;\;i=1,2,\cdots ,n\\ & & \sum_{i=1}^{n}\omega_{i}=1. \end{array}$$ (19)

For example, when *ω* = (1, 0, 0, …, 0), *F*(*a*_1_, *a*_2_, …, *a*_*n*_) = *max*(*a*_1_, *a*_2_, …, *a*_*n*_) = *b*_1_, OWA operator is equal to the “or” operator in fuzzy operation. Or when $\omega =(\frac{1}{n},\frac{1}{n},\cdots ,\frac{1}{n})$, OWA operator is equivalent to arithmetic mean operator now.

## Generalized interval correlation coefficient

Interval correlation coefficient, which describe the degree of correlation between evidence, is able to measure evidence conflict. But as the status of problems in the open world becomes more and more important, interval correlation coefficient as a definition of measurement method in the closed world, hindering the application and development of it. In this paper, generalized interval correlation coefficient *gir* is proposed, which can manage open world problems.

Let *m* be a GBPA on the frame of discernment Θ = {∅, *θ*_1_, *θ*_2_, …, *θ*_*N*_}. It is expressed as the confidence interval of all the single subset of elements on the frame of discernment. That is, *m* is expressed as

$$\begin{array}{c} \{\left[GBel\left(\emptyset \right),GPl\left(\emptyset \right)\right],\left[GBel\left(\theta_{1}\right),GPl\left(\theta_{1}\right)\right],\left[GBel\left(\theta_{2}\right),GPl\left(\theta_{2}\right)\right],\cdots ,\left[GBel\left(\theta_{N}\right),GPl\left(\theta_{N}\right)\right]\}. \end{array}$$ (20)

We use the interval information on the belief function which is defined in Eq (10) and the plausibility function which is defined in Eq (11) of propositions in GET. The confidence interval of the single subset *θ* is expressed as [*Bel*(*θ*), *Pl*(*θ*)]. *Bel*(*θ*) indicates the part of the evidence that explicitly supports *θ*, and *Pl*(*θ*) indicates the part of the evidence that potential supports *θ*. The actual trust value of *θ* should be in [*Bel*(*θ*), *Pl*(*θ*)].

The simulation consequence shows that calculating the interval of entire 2^Θ^ subsets not only resulted in a huge computational quantity, but also caused double counting of information. For instance, both *GPl*(*a*) and *GPl*(*a*, *c*) contain the information of *m*(*a*), however our purpose is assigning *m*(*a*) to only one monad because the information containing in *m*(*a*) is definite. Utilizing only the value of the single subset can preserve the vast majority of valid information while having a simpler calculation.

In classic D-S theory, the mass function and its interval representation are equivalent. The mass function can be calculated by its interval representation as follows [59],

$$\begin{array}{c} m\left(A\right)=\sum_{B\subseteq A}{\left(-1\right)}^{\left|A|-|B\right|}Bel\left(A\right) \end{array}$$ (21)

this formula is from Möbius Inversion Theorem, and the term (−1)^|*A*|−|*B*|^ actually mirrors the mutual inclusion of the subsets. Compared to classical evidence theory, GBPA eliminates the *m*(∅) = 0 constraint. The interval representation of *m*(∅) happens to be [*m*(∅), *m*(∅)] since *GBel*(∅) = *GPl*(∅) = *m*(∅), and the interrelationships between non-empty sets in GET is the same as above. Therefore GBPA can be obtained from its interval representation by the same method, on the contrary it is clearly that the interval representation can be calculated with GBPA. So we take into account the interval [*GBel*(*θ*), *GPl*(*θ*)] to reserve the valuable information as much as possible. The following example illustrates the specific calculation method.

*Example* 1. Suppose that there is a frame of discernment of Θ = {*a*, *b*, *c*}, and a GBPA is given as

$$\begin{array}{c} m(\emptyset )=0.1;m(a)=0.2;m(a,b)=0.4;m(b,c)=0.3. \end{array}$$

First need to get the *GBel* and *GPl* of all the single subset

$$\begin{array}{c} GBel(\emptyset )=0.1;GBel(a)=0.2;GBel(b)=0;GBel(c)=0, \end{array}$$

$$\begin{array}{c} GPl(\emptyset )=0.1;GPl(a)=0.6;GPl(b)=0.7;GPl(c)=0.3. \end{array}$$

Then, the confidence interval representation of *m* is expressed as

$$\begin{array}{c} \{[0.1,0.1],[0.2,0.6],[0,0.7],[0,0.3]\}. \end{array}$$

**Definition 1**. Let *m*_1_ and *m*_2_ be two GBPAs on the same frame of discerment Θ = {*θ*_1_, *θ*_2_, …, *θ*_*N*_}, the relevance between *m*_1_ and *m*_2_ is defined as

$$\begin{array}{c} c\left(m_{1},m_{2}\right)=\frac{1}{2}\left(Bel_{1}\left(\emptyset \right)Bel_{2}\left(\emptyset \right)+Pl_{1}\left(\emptyset \right)Pl_{2}\left(\emptyset \right)+\sum_{i=1}^{N}\left(Bel_{1}\left(\theta_{i}\right)Bel_{2}\left(\theta_{i}\right)+Pl_{1}\left(\theta_{i}\right)Pl_{2}\left(\theta_{i}\right)\right)\right). \end{array}$$ (22)

**Definition 2**. Let *m*_1_ and *m*_2_ be two GBPAs on the same frame of discernment Θ = {*θ*_1_, *θ*_2_, …, *θ*_*N*_}, the generalized interval correlation coefficient *gir* is defined as

$$\begin{array}{c} gir\left(m_{1},m_{2}\right)=\frac{c(m_{1},m_{2})}{\sqrt{c\left(m_{1},m_{1}\right)\times c\left(m_{2},m_{2}\right)}}, \end{array}$$ (23)

The generalized interval correlation coefficient *gir* has the following three properties.

1. *gir*(*m*_1_, *m*_2_) = 1, when *m*_1_ is equal to *m*_2_;
2. *gir*(*m*_1_, *m*_2_) = *gir*(*m*_2_, *m*_1_);
3. 0 ≤ *gir*(*m*_1_, *m*_2_)≤1.

When *m*_1_(∅) = 0 and *m*_2_(∅) = 0, i.e., *m*_1_ and *m*_2_ are two BPAs in closed world, Eq (22) is same to Eq (17), and it is clear that Eqs (23) and (16) are also identical. Therefore, the *gir* degenerates to the interval correlation coefficient when FOD is complete.

In the following, some examples illustrate that *gir* can measure the conflict between GBPAs in open world.

*Example* 2. Assume a frame of discernment Θ = {*a*, *b*, *c*}, and two GBPAs are given as

$$\begin{array}{c} m_{1}\left(\emptyset \right)=0.1;m_{1}\left(a\right)=0.35;m_{1}\left(a,b\right)=0.55, \end{array}$$

$$\begin{array}{c} m_{2}\left(\emptyset \right)=0.5;m_{2}\left(b,c\right)=0.4;m_{2}\left(c\right)=0.1. \end{array}$$

In this example, we should first denote *m*_1_ and *m*_2_ in the form of the confidence interval of all the single subset of elements on the FOD. In order to express more intuitively, we use Table 1 to get the confidence interval.

**Table 1:** *Bel*(*θ*_*i*_) and *Pl*(*θ*_*i*_) for *m*_1_ and *m*_2_ in Example 2.

single subset *θ*_*i*_	*Bel*_1_(*θ*_*i*_)	*Pl*_1_(*θ*_*i*_)	*Bel*_2_(*θ*_*i*_)	*Pl*_2_(*θ*_*i*_)
∅	0.1	0.1	0.5	0.5
*a*	0.35	0.9	0	0
*b*	0	0.55	0	0.4
*c*	0	0	0.1	0.5

And *gir* is calculated as follows,

$$\begin{array}{ccc} c(m_{1},m_{2}) & = & \frac{1}{2}(Bel_{1}\left(\emptyset \right)Bel_{2}\left(\emptyset \right)+Pl_{1}\left(\emptyset \right)Pl_{2}\left(\emptyset \right)\\ & & +\sum_{i=1}^{3}\left(Bel_{1}\left(\theta_{i}\right)Bel_{2}\left(\theta_{i}\right)+Pl_{1}\left(\theta_{i}\right)Pl_{2}\left(\theta_{i}\right)\right))\\ & = & \frac{1}{2}(0.1\times 0.5+0.1\times 0.5+0.35\times 0+0.9\times 0\\ & & +0\times 0+0.55\times 0.4+0\times 0.1+0\times 0.5)\\ & = & 0.16. \end{array}$$ (24)

$$\begin{array}{ccc} c(m_{1},m_{1}) & = & \frac{1}{2}(Bel_{1}\left(\emptyset \right)Bel_{1}\left(\emptyset \right)+Pl_{1}\left(\emptyset \right)Pl_{1}\left(\emptyset \right)\\ & & +\sum_{i=1}^{3}\left(Bel_{1}\left(\theta_{i}\right)Bel_{1}\left(\theta_{i}\right)+Pl_{1}\left(\theta_{i}\right)Pl_{1}\left(\theta_{i}\right)\right))\\ & = & \frac{1}{2}(0.1\times 0.1+0.1\times 0.1+0.35\times 0.35+0.9\times 0.9\\ & & +0\times 0+0.55\times 0.55+0\times 0+0\times 0)\\ & = & 0.6275. \end{array}$$ (25)

$$\begin{array}{ccc} c(m_{2},m_{2}) & = & \frac{1}{2}(Bel_{2}\left(\emptyset \right)Bel_{2}\left(\emptyset \right)+Pl_{2}\left(\emptyset \right)Pl_{2}\left(\emptyset \right)\\ & & +\sum_{i=1}^{3}\left(Bel_{2}\left(\theta_{i}\right)Bel_{2}\left(\theta_{i}\right)+Pl_{2}\left(\theta_{i}\right)Pl_{2}\left(\theta_{i}\right)\right))\\ & = & \frac{1}{2}(0.5\times 0.5+0.5\times 0.5+0\times 0+0\times 0\\ & & +0\times 0+0.4\times 0.4+0.1\times 0.1+0.5\times 0.5)\\ & = & 0.46. \end{array}$$ (26)

$$\begin{array}{ccc} gir(m_{1},m_{2}) & = & \frac{c(m_{1},m_{2})}{\sqrt{c\left(m_{1},m_{1}\right)\times c\left(m_{2},m_{2}\right)}}\\ & = & \frac{0.16}{\sqrt{0.6275\times 0.46}}=0.298. \end{array}$$ (27)

It is obvious that the traditional interval correlation coefficient does not apply to the problems in this circumstance, because *m*_1_ and *m*_2_ are not BPAs but GBPAs. Using *gir* as defined in this paper, the degree of correlation between the two pieces of evidence can be calculated, which is able to determine whether the evidence are in high conflict according to the specific situation and whether it is appropriate to use generalized combination rule.

*Example* 3. Let a frame of discernment be Θ = {*a*, *b*}, six sets of GBPAs and their *gir* and *k* are given in Table 2.

**Table 2:** GBPAs and their *gir* and *k* in Example 3.

GBPAs	*gir*	*k*
*m*_1_(*a*) = 0.5, *m*_1_(∅) = 0.5	1	0.75
*m*_2_(*a*) = 0.5, *m*_2_(∅) = 0.5		
*m*_1_(*a*) = 0.6, *m*_1_(∅) = 0.4	0.923	0.76
*m*_2_(*a*) = 0.4, *m*_2_(∅) = 0.6		
*m*_1_(*a*) = 0.7, *m*_1_(∅) = 0.3	0.724	0.79
*m*_2_(*a*) = 0.3, *m*_2_(∅) = 0.7		
*m*_1_(*a*) = 0.8, *m*_1_(∅) = 0.2	0.471	0.84
*m*_2_(*a*) = 0.2, *m*_2_(∅) = 0.8		
*m*_1_(*a*) = 0.9, *m*_1_(∅) = 0.1	0.220	0.91
*m*_2_(*a*) = 0.1, *m*_2_(∅) = 0.9		
*m*_1_(*a*) = 1, *m*_1_(∅) = 0	0	1
*m*_2_(*a*) = 0, *m*_2_(∅) = 1		

**Fig 1:**
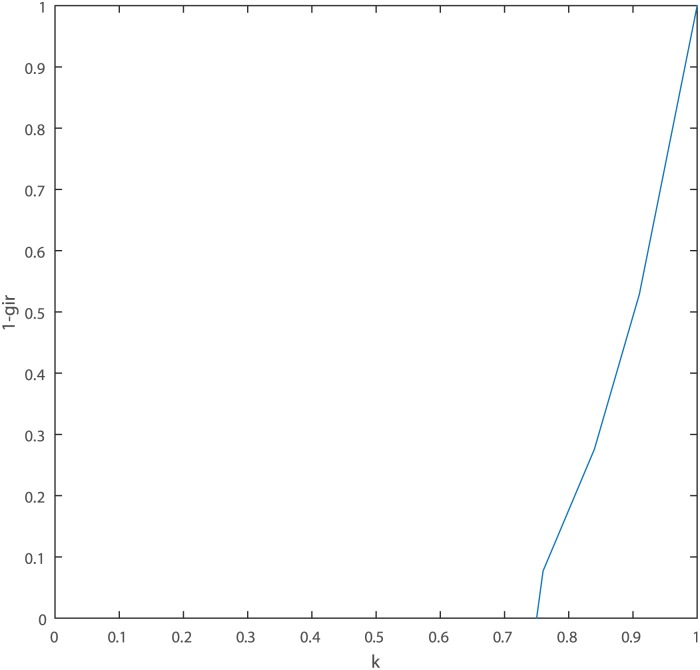
The relationship between *k* and 1 − *gir* in Example 3.

As shown in Table 2, *gir* is decreasing monotone and can be used to measure the degree of conflict between GBPAs. The following Fig 1 graphically show the positive correlation between 1 − *gir* and *k*. Moreover, *k* is unconventionally large because of its defects on dealing with the empty set yet 1 − *gir* changes smoothly and evenly from 0 to 1. The establishment of above three properties and this example indicate that the generalized interval correlation coefficient *gir* is a measure of the consistency between two GBPAs. The larger *gir*, the greater the correlation between the two evidence, so the smaller the conflict between them, and vice versa.

## A combinatorial parameter based on OWA operator

Since Zadeh [40] proposed that Dempster’s combination rule in highly conflict evidence would produce a perverse result, many scholars had tried to solve the problem from various angles. Before evidence fusion, the degree of conflict between the evidence must be measured first. How to obtain an effective method to measure the conflict between the evidence, and based on the conflict measurement to determine whether the evidence required for processing prior to combination, has become an essential issue.

So far, there have been several methods to measure the evidence conflict in open world. For example, generalized conflict coefficient *k* mainly indicates the degree of the two evidence contained in each other, generalized evidence distance *dis* can calculate the difference between the evidence, generalized interval correlation coefficient *gir* is proposed to measure the correlation between the evidence. The above three coefficients measure the evidence conflict from three different aspects. In order to achieve a comprehensive conflict measure from multiple aspects, a combining parameter based on OWA operator is proposed in this paper. When one or two coefficients are invalid, the combinatorial parameter can still effectively measure the evidence conflict in open world.

**Definition 3**. The ordered weighted vector [60] *ω* = (*ω*_1_, *ω*_2_, …, *ω*_*n*_) is determined by the following equation:

$$\begin{array}{c} \omega_{i}=Q\left(\frac{i}{n}\right)-Q\left(\frac{i-1}{n}\right),\;\;i=1,2,\cdots ,n \end{array}$$ (28)

where *Q* is called fuzzy semantic quantization operator, and satisfies

$$\begin{array}{c} Q\left(m\right)=\{\begin{array}{cc} 0 & m<\alpha ,\\ \frac{m-\alpha }{\beta -\alpha } & \alpha \leq m\leq \beta ,\\ 1 & m>\beta . \end{array} \end{array}$$ (29)

*α* and *β* are defined by the fuzzy semantics in the specific case and satisfy *α*, *β* ∈ [0, 1]. The variable *α* and *β* of the three common situation “as much as possible”, “at least half”, “great majority” are respectively (0.5, 1), (0, 0.5), (0.3, 0.8).

*Example* 4. Suppose there are five ordered coefficients *a*, *b*, *c*, *d*, *e*, use the “great majority” rule in the OWA operator, i.e., now *n* = 5, *α* = 0.3 and *β* = 0.8.

So,

$$\begin{array}{c} Q\left(m\right)=\{\begin{array}{cc} 0 & m<0.3,\\ \frac{m-0.3}{0.5} & 0.3\leq m\leq 0.8,\\ 1 & m>0.8. \end{array} \end{array}$$ (30)

The calculate the process of *ω* is shown in Table 3.

**Table 3:** *ω*_*i*_ in Example 4.

*i*	$\frac{i}{n}$	$\frac{i-1}{n}$	$Q(\frac{i}{n})$	$Q(\frac{i-1}{n})$	$\omega_{i}=Q\left(\frac{i}{n}\right)-Q\left(\frac{i-1}{n}\right)$
1	0.2	0	0	0	0
2	0.4	0.2	0.2	0	0.2
3	0.6	0.4	0.6	0.2	0.4
4	0.8	0.6	1	0.6	0.4
5	1	0.8	1	1	0

From Table 3, we can get the OWA operator *ω* = (0, 0.2, 0.4, 0.4, 0), and *OWA*(*a*, *b*, *c*, *d*, *e*) = 0.2 × *b* + 0.4 × *c* + 0.4 × *d*.

When using OWA operator weighting, the information characterization of each parameter must be unified first. Three parameters are unified into the larger the parameter, the greater the conflict, so calculate with (1 − *gir*) instead of *gir*. In this paper, these three parameters *k*, *dis*, and (1 − *gir*) are weighted by the OWA operator, and we adopt the fuzzy semantics of “at least half” (0, 0.5) to retain more information in the variable. When *n* = 3, *α* = 0 and *β* = 0.5, it is easy to get the ordered weighted vector $\omega^{*}=\left(\omega_{1}^{*},\omega_{2}^{*},\omega_{3}^{*}\right)=\left(\frac{2}{3},\frac{1}{3},0\right)$ from Eqs (28) and (29). The combinatorial conflict parameter *comb* is defined as follows.

**Definition 4**. Let *m*_1_ and *m*_2_ be two GBPAs on the same frame of discernment Θ, and calculate *k*, *dis*, and (1 − *gir*). The combinatorial conflict parameter *comb* is defined as

$$\begin{array}{c} comb=OWA\left(k,dis,1-gir\right)=\sum_{i=1}^{3}\omega_{i}^{*}a_{i}, \end{array}$$ (31)

where $\omega^{*}=\left(\frac{2}{3},\frac{1}{3},0\right)$, and *a*_*i*_ is the i-th largest element in array (*k*, *dis*, 1 − *gir*).

Deng [52] proposed a conflict model for GET, denoted as

$$\begin{array}{c} cf_{G}\left(m_{1},m_{2}\right)=<K,dis>. \end{array}$$ (32)

The objective of the proposed model is to offer to the decision maker a two-tuples conflict measurement. It also considers two different parameters and has a number of robustness. However, in practical engineering application, we prefer a determinate number rather than a two-tuples model, since it must be analyzed first the respective applicable occasions of each coefficient in the two-dimensional model, which greatly reduces the efficiency. Moreover, as Deng’s proposal, when the FOD is incomplete and *m*(∅) ≠ 0, the conflict model should mainly consider *K*, whereas it had better depend on *dis*. GET as the extension of D-S theory, its biggest advantage and innovation is capable of managing the problems in open world, yet the processing method of its conflict model appears a recession since many problems in open world are regarded as in the closed world.

In the following, some numerical examples are given to demonstrate the OWA operator calculation process and its superiority.

*Example* 5. Suppose that there is a frame of discernment of Θ = {*a*, *b*, *c*}, and two GBPAs are given as

$$\begin{array}{c} m_{1}\left(a\right)=0.99;m_{1}\left(b\right)=0.01, \end{array}$$

$$\begin{array}{c} m_{2}\left(b\right)=0.01;m_{2}\left(c\right)=0.99. \end{array}$$

We can compute

$$\begin{array}{c} k=0.9999,\;dis=0.99,\;1-gir=0.9999 \end{array}$$

so

$$\begin{array}{ccc} comb & = & OWA(k,dis,1-ir)\\ & = & \sum_{i=1}^{3}\omega_{i}^{*}a_{i}\\ & = & \frac{2}{3}\times k+\frac{1}{3}\times \left(1-gir\right)+0\times dis\\ & = & 0.9999 \end{array}$$ (33)

This example is Zadeh’s classic paradox, the direct combination of these two GBPAs through the Dempster’s combination rule will produce results contrary to common sense. The new combinatorial parameter *comb* in this example is very close to 1, which indicates there is a quite high degree of conflict between two evidence, and not suitable for Dempster’s rule in this case.

*Example* 6. Assume a frame of discernment Θ = {*a*, *b*, *c*}, and that two GBPAs are given as

$$\begin{array}{ccc} m_{1}\left(a\right) & = & 0.25;m_{1}\left(a,b\right)=0.75,\\ m_{2}\left(a\right) & = & 0.15;m_{2}\left(a,c\right)=0.85. \end{array}$$

We first calculate such that

$$\begin{array}{c} k=0,\;dis=0.656,\;1-gir=0.384, \end{array}$$

and

$$\begin{array}{ccc} comb & = & \frac{2}{3}\times dis+\frac{1}{3}\times \left(1-gir\right)+0\times k\\ & = & 0.565 \end{array}$$ (34)

In addition, Lefèvre [47] proposed an interesting method to measure the conflict of evidence. He ingeniously combined the information of conflict coefficient and evidence distance by weighted average, and regarded the mass assigned to conflict, that is *m*(∅) as an alarm signal announcing disagreement. However, the essence of Lefèvre’s method is an adaptive weighting between two combination methods which have the completely opposite allocation rule about *m*(∅), namely *m*(∅) = 0 in Dempster’s combination rule while it is approximately 1 in conjunctive rule. The evidence distance plays a weight role to command the result on the basis of the conflict coefficient *k*. Therefore, as long as the *m*(∅) in conjunctive rule is small, videlicet *k* is close to 0, the conflict management results will be incorrect just like this example which shows the evidences are totally identical. Based on the above analysis, our method *comb* is obviously more reasonable.

The evidence in this case is relatively easy to appear in the application. For instance, the frame of discernment of the multi-sensor contains two very similar units *b* and *c* in military reconnaissance, the information obtained by sensors has a high probability of confusing *b* and *c* with malicious interference from the enemy. If the conflict between the evidence is measured by conflict coefficient *k*, the result is 0. And at this time using Dempster’s combination rule will get the fallacious result *m*(*a*) = 1, which indicates the information may be unable to identify *b* and *c*. From the results, *m*_1_ and *m*_2_ are conflicting evidence of each other, and must be carefully combined. In this case directly using the combination rule to deal with the GBPAs may lead to an erroneous result, and should try to modify the evidence model first.

*Example* 7. Let a frame of discernment be Θ = {*a*, *b*, *c*}, and consider two GBPAs defined as

$$\begin{array}{ccc} m_{1}\left(\emptyset \right) & = & 0.1;m_{1}\left(a\right)=0.35;m_{1}\left(a,b\right)=0.55,\\ m_{2}\left(\emptyset \right) & = & 0.5;m_{2}\left(b,c\right)=0.4;m_{2}\left(c\right)=0.1. \end{array}$$

According to the results of Example 2, *gir* between *m*_1_ and *m*_2_ is 0.298,

$$\begin{array}{ccc} dis & = & \sqrt{\frac{1}{2}{\left(\vec{m_{1}}-\vec{m_{2}}\right)}^{T}\bar{D}\left(\vec{m_{1}}-\vec{m_{2}}\right)}\\ & = & \sqrt{\frac{1}{2}\left(-0.4,0.35,-0.1,0.55,-0.4\right)\left(\begin{array}{ccccc} 1 & 0 & 0 & 0 & 0\\ 0 & 1 & 0 & \frac{1}{2} & 0\\ 0 & 0 & 1 & \frac{1}{2} & 0\\ 0 & \frac{1}{2} & 0 & 1 & \frac{1}{3}\\ 0 & 0 & \frac{1}{2} & \frac{1}{3} & 1 \end{array}\right)\left(\begin{array}{c} -0.4\\ 0.35\\ -0.1\\ 0.55\\ -0.4 \end{array}\right)}=0.648 \end{array}$$ (35)

and we can calculate that *k* = 0.78, 1 − *gir* = 0.702, so

$$\begin{array}{ccc} comb & = & \frac{2}{3}\times k+\frac{1}{3}\times \left(1-gir\right)+0\times dis\\ & = & 0.754 \end{array}$$ (36)

Obviously, *comb* is able to effectively measure the conflict between two GBPAs in open world.

*Example* 8. We consider a frame of discernment Θ = {*a*, *b*, *c*} and two GBPAs are

$$\begin{array}{c} m_{1}\left(a\right)=0.5;m_{1}\left(c\right)=0.5, \end{array}$$

$$\begin{array}{c} m_{2}\left(b\right)=0.5;m_{2}\left(\emptyset \right)=0.5. \end{array}$$

And we calculate that *k* = 1, *dis* = 0.707 and 1 − *gir* = 1, so *comb* = 1. In such a case, *dis* is relatively out of operation which is not the case with our approach *comb*.

**Fig 2:**
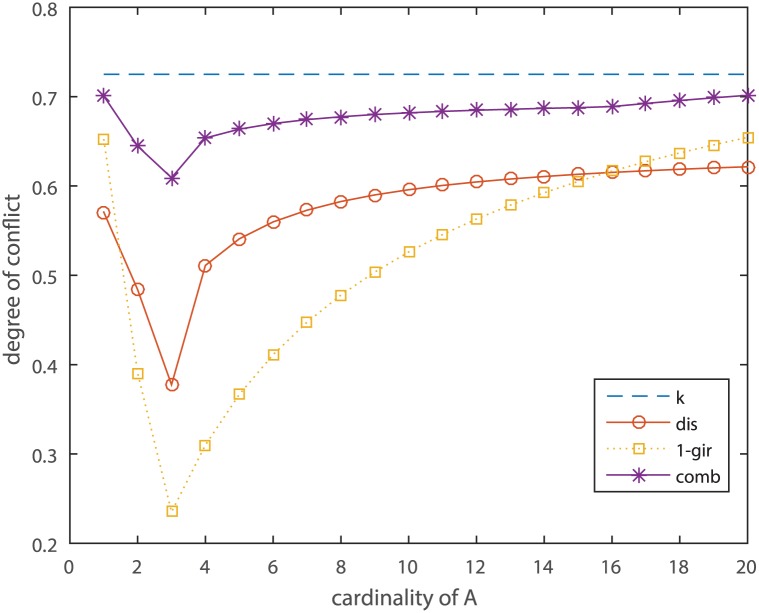
Comparison of *comb* with the old conflict coefficients in Example 9.

*Example* 9. Suppose that we have a frame of discernment of Θ = {1, 2, 3, …, 20}, and two GBPAs *m*_1_ and *m*_2_ are defined as

$$\begin{array}{cc} & m_{1}\left(\emptyset \right)=0.45;m_{1}\left(1,2,3\right)=0.55,\\ & m_{2}\left(\emptyset \right)=0.1;m_{2}\left(A\right)=0.5;m_{2}\left(4\right)=0.4, \end{array}$$

where *A* is a varying subset of Θ. *A* increments one more element each time, starting at *A* = {1}, and ending with *A* = {1, 2, 3, …, 20}. The *comb* and the old coefficients between *m*_1_ and *m*_2_ are shown in Table 4 and Fig 2.

**Table 4:** Comparison of *comb* with the old conflict coefficients in Example 9.

Cases	*k*	*dis*	1 − *gir*	*comb*
A = {1}	0.725	0.5708	0.6524	0.7008
A = {1, 2}	0.725	0.4839	0.3905	0.6446
A = {1, 2, 3}	0.725	0.3775	0.2350	0.6092
A = {1, 2, …, 4}	0.725	0.5111	0.3100	0.6537
A = {1, 2, …, 5}	0.725	0.5408	0.3666	0.6636
A = {1, 2, …, 6}	0.725	0.5598	0.4112	0.6699
A = {1, 2, …, 7}	0.725	0.5729	0.4475	0.6743
A = {1, 2, …, 8}	0.725	0.5826	0.4779	0.6775
A = {1, 2, …, 9}	0.725	0.5900	0.5037	0.6800
A = {1, 2, …, 10}	0.725	0.5958	0.5261	0.6819
A = {1, 2, …, 11}	0.725	0.6006	0.5456	0.6835
A = {1, 2, …, 12}	0.725	0.6045	0.5630	0.6848
A = {1, 2, …, 13}	0.725	0.6078	0.5785	0.6859
A = {1, 2, …, 14}	0.725	0.6106	0.5924	0.6869
A = {1, 2, …, 15}	0.725	0.6131	0.6051	0.6877
A = {1, 2, …, 16}	0.725	0.6152	0.6166	0.6889
A = {1, 2, …, 17}	0.725	0.6170	0.6272	0.6924
A = {1, 2, …, 18}	0.725	0.6187	0.6370	0.6957
A = {1, 2, …, 19}	0.725	0.6202	0.6460	0.6987
A = {1, 2, …, 20}	0.725	0.6215	0.6544	0.7015

Table 4 and Fig 2 show that, regardless of how the subset *A* changes, the value of *k* remains constant, which is obviously contrary to common sense. Although *dis* and (1 − *gir*) vary with the subset *A*, due to lopsided processing of the empty set, the value of *dis* and (1 − *gir*) is too small when *A* = {1, 2, 3} or in its vicinity. The new combinatorial parameter *comb* correctly reflects that the conflict between *m*_1_ and *m*_2_ is minimal when *A* = {1, 2, 3}, and the greater the deviation of the subset *A* from {1, 2, 3}, the greater the conflict of evidence. Furthermore, the value of *comb* is also intuitive.

In summary, three existing measurements *k*, *dis* and 1 − *gir* sometimes produce counter-intuitive and inaccurate results. This does not explain that they are worthless, but is actually quite the opposite. Three coefficient measure the conflict from different aspects, that is, k mainly calculates the degree of the two evidence contained in each other, dis can indicate the difference between the evidence, and gir is proposed to measure the correlation between the evidence. *comb* is a synthesis of the three coefficients, it blends various effective information and avoids the errors caused by the one-sidedness of single factor, accordingly is highly accurate.

## Conclusion

In the application of information fusion, GET has been extensively used due to its ability to deal with evidence conflict when the frame of discernment is incomplete. But how to determine a valid parameter to measure the degree of conflict between the evidence in open world is still an open issue. We first extend the interval correlation coefficient to *gir* in this paper, which is capable of calculating the correlation between the evidence in incomplete FOD.

D-S theory and GET are the methods of processing information fusion, the parameters describing the evidence conflict are also a kind of information. So we use the “information fusion” idea to combine the three coefficients. By extracting their respective valid information, we obtain a combinatorial parameter based on OWA operator to measure the evidence conflict. Several numerical examples demonstrate that compared with using a single parameter, the proposed method is more reliable and effective.
